# Extreme Sensory Complexity Encoded in the 10-Megabase Draft Genome Sequence of the Chromatically Acclimating Cyanobacterium *Tolypothrix* sp. PCC 7601

**DOI:** 10.1128/genomeA.00355-15

**Published:** 2015-05-07

**Authors:** Shaila Yerrapragada, Animesh Shukla, Kymberlie Hallsworth-Pepin, Kwangmin Choi, Aye Wollam, Sandra Clifton, Xiang Qin, Donna Muzny, Sriram Raghuraman, Haleh Ashki, Akif Uzman, Sarah K. Highlander, Bartlomiej G. Fryszczyn, George E. Fox, Madhan R. Tirumalai, Yamei Liu, Sun Kim, David M. Kehoe, George M. Weinstock

**Affiliations:** a Human Genome Sequencing Center, Baylor College of Medicine, Houston, Texas, USA; b Department of Biology, Indiana University, Bloomington, Indiana, USA; c The Genome Institute, Washington University in St. Louis, St. Louis, Missouri, USA; d School of Informatics, Indiana University, Bloomington, Indiana, USA; e College of Sciences and Technology, University of Houston-Downtown, Houston, Texas, USA; f Department of Biology and Biochemistry, University of Houston, Houston, Texas, USA

## Abstract

*Tolypothrix* sp. PCC 7601 is a freshwater filamentous cyanobacterium with complex responses to environmental conditions. Here, we present its 9.96-Mbp draft genome sequence, containing 10,065 putative protein-coding sequences, including 305 predicted two-component system proteins and 27 putative phytochrome-class photoreceptors, the most such proteins in any sequenced genome.

## GENOME ANNOUNCEMENT

Cyanobacteria have a tremendous capacity to acclimate to changing environments (1). The filamentous cyanobacterium *Tolypothrix* sp. PCC 7601 is studied for its phenotypic plasticity in changing environments (2). Isolated from a Connecticut lake and named *Fremyella diplosiphon* (UTEX 481), it was added to the Pasteur Culture Collection as *Calothrix* sp. PCC 7601 and renamed *Tolypothrix* sp. PCC 7601. It is a model organism for studying the mechanism and regulation of chromatic acclimation, the reversible modification of photosynthetic light-harvesting antennae in response to changes in ambient light color, shifting the cell phenotype between red and blue-green (2–4).

*Tolypothrix* sp. PCC 7601 responds to many additional abiotic conditions. It acclimates to low sulfate conditions by producing antennae proteins depleted in sulfur-containing amino acids (5, 6). It has multiple developmental pathways, changes its cellular morphology and average filament length in different ambient light colors, and historically could reduce atmospheric nitrogen (7). Thus, it possesses an extensive repertoire of environmental responses. Its genome contains large numbers of genes encoding regulatory components, particularly two-component system proteins.

Shortened filament mutant SF33 (also called Fd33) was generated from *F. diplosiphon* (UTEX 481) (8) and used for genome sequencing. The sequence was generated using a full 3-kb paired-end Titanium 454 sequencing run, representing 46.6-fold genome coverage. A Newbler draft assembly was generated using Newbler version vMapAsmResearch-02/17/2010. The draft genome is 9,963,861 bp in length and contains 157 contigs (>139 bp in length) with a mean contig size of 63,464 bp and a maximum length of 955,511 bp. The draft was not further joined due to the presence of approximately 150 repetitive regions (probable endogenous transposable elements). The mean G+C genome content is 40.6%. Annotation and gene prediction used the TIGR Gene Indices gene annotation process (9). Coding sequences were predicted using GeneMark (10) and Glimmer3 (11). Intergenic regions not spanned by GeneMark and Glimmer3 were blasted against NCBI’s nonredundant bacterial (NR) database. Loci were then defined by clustering predictions with the same reading frame. The best prediction at each locus was selected by evaluating all predictions against nonredundant bacterial, NR, and Pfam evidence (12) and resolving overlaps between adjacent coding genes. tRNA genes were determined using tRNAscan-SE (13) and noncoding RNA genes by RNAmmer (14) and Rfam (15). The final gene set was processed through KEGG (16), psortB (17), and Interproscan (18) to determine possible function. Gene product names were determined by BLAST Extend Repraze (http://sourceforge.net/projects/ber/).

A total of 10,065 coding sequences were predicted, including 305 two-component-system proteins and 27 phytochrome-class photoreceptors, the largest number of each of these groups of sensory proteins reported for any bacterial genome to date. These results suggest the presence of complex sensory and regulatory systems that are required for the extensive environmental responsiveness and phenotypic plasticity of this cyanobacterium.

These sequence data will be useful for elucidating the regulatory systems of prokaryotes with large, complex genomes.

### Nucleotide sequence accession numbers.

This whole-genome shotgun project has been deposited in DDBJ/EMBL/GenBank under the accession number AGCR00000000. The version described in this paper is the first version, AGCR01000000.
